# Plasma-Coated Collagen Membranes Gain Barrier Function Through Heat Treatment

**DOI:** 10.3390/jfb17020095

**Published:** 2026-02-14

**Authors:** Karol Ali Apaza Alccayhuaman, Patrick Heimel, Stefan Lettner, Richard J. Miron, Carina Kampleitner, Layla Panahipour, Ulrike Kuchler, Reinhard Gruber

**Affiliations:** 1 Department of Oral Biology, University Clinic of Dentistry, Medical University of Vienna, 1090 Vienna, Austria; 2 Karl Donath Laboratory for Hard Tissue and Biomaterial Research, University Clinic of Dentistry, Medical University of Vienna, 1090 Vienna, Austria; 3 Department of Periodontology, School of Dental Medicine, University of Bern, 3010 Bern, Switzerland; 4 Biomaterials and Technology, Department Research, University Center for Dental Medicine Basel UZB, University of Basel, 4058 Basel, Switzerland; 5 Austrian Cluster for Tissue Regeneration, 1200 Vienna, Austria; 6 Ludwig Boltzmann Institute for Traumatology, The Research Center in Cooperation with AUVA, 1200 Vienna, Austria; 7 Department of Oral Surgery, University Clinic of Dentistry, Medical University of Vienna, 1090 Vienna, Austria

**Keywords:** guided bone regeneration, collagen membrane, platelet-poor plasma, thermal denaturation, membrane stability, bone regeneration, micro–computed tomography, histology

## Abstract

Guided bone regeneration (GBR) relies on barrier membrane integrity to prevent soft-tissue ingrowth. Although collagen membranes are widely used, their limited longevity can compromise space maintenance, underscoring the need for strategies that enhance membrane stability without impairing the regenerative potential. We hypothesized that thermal denaturation of platelet-poor plasma (PPP), combined with heat-induced modifications of collagen fibrils, could generate a volume-stable, plasma-rich composite that preserves membrane structure and restricts cellular penetration. To test this proof-of-principle concept, collagen membranes were soaked in PPP and either kept at room temperature or subjected to thermal treatment (75 °C/10 min) prior to implantation in rat calvarial defects. Bone regeneration and membrane behavior were evaluated after three weeks using micro-computed tomography (micro-CT) and histology. Micro-CT suggested only minor numerical differences in mineralized tissue between groups; however, these data should not be overinterpreted because micro-CT cannot differentiate mineralization formed within the collagen membrane from mineralization adjacent to it. Consistent with this limitation, histology demonstrated that mineral deposition and early bone formation extended into the structure of room-temperature PPP membranes, whereas mineralized tissue in the thermally treated group was predominantly located outside the membrane, indicating reduced osteoconductive integration within the membrane. Together, these findings support that thermal denaturation of PPP shifts early composite membrane behavior toward barrier-dominant characteristics at the expense of intramembranous mineralization.

## 1. Introduction

Blood is a complex biological fluid composed of cellular elements suspended in liquid plasma; however, upon injury, coagulation transforms circulating plasma into a transient fibrin matrix that stabilizes the wound and temporarily fills the defect space [1]. During this process, fibrinogen molecules polymerize into an insoluble network that entraps cells, forming a scaffold through which inflammatory and progenitor cells migrate [2,3]. Subsequent fibrinolysis enables vascular ingrowth and the arrival of osteogenic cells, making the quality and degradability of the provisional matrix a key determinant of early bone regeneration [4,5,6].

To enhance these natural regenerative processes, autologous platelet concentrates such as platelet-rich plasma (PRP) and platelet-rich fibrin (PRF) have been introduced [7,8]. By depleting erythrocytes while selectively concentrating platelets and leukocytes, these preparations generate fibrin matrices that release bioactive molecules and support angiogenesis and cell recruitment. PRF membranes have therefore gained wide clinical use in alveolar ridge preservation, periodontal regeneration, guided bone regeneration (GBR) [9,10,11], sinus floor elevation, and the management of chronic ulcers [12,13]. However, similar to native fibrin clots, conventional PRF degrades rapidly—typically within two weeks—thereby limiting its ability to maintain space during GBR procedures that require mechanical stability over longer healing periods [12].

Recently, thermal modification of plasma components has emerged as an alternative strategy to increase the stability of fibrin-based biomaterials [13]. Plasma proteins—including fibrinogen, albumin, and globulins—denature at characteristic temperatures [14,15], with albumin exhibiting major thermal transitions at approximately 63–72 °C [16]. Heating induces irreversible coagulation of these proteins and may also reduce the activity of heat-resistant molecules such as plasmin [17]. Because heat-coagulated albumin is not readily degraded by physiological endocytic pathways [18], thermally treated plasma undergoes a transition from a soluble phase to a semi-solid, volume-stable matrix—analogous to the denaturation of egg white during cooking.

Based on the principle of heat-denatured plasma, it has been proposed that thermal treatment of platelet-poor plasma (PPP) at 75 °C for 10 min induces denaturation of plasma proteins, which can subsequently be combined with a non-heated buffy coat to form albumin-based platelet-rich fibrin (Alb-PRF) or extended PRF (e-PRF) [13,19,20]. Notably, Alb-PRF and e-PRF—but not conventional leukocyte-PRF or horizontal PRF—exhibit sustained volume stability following subcutaneous implantation [21]. Heating of fibrinogen and PRF to prepare a matrix was already proposed as a strategy to delay degradation [15,22]. Although this transformation preserves membrane structure, the resulting protein coagulum is likely to impede cellular infiltration and remodeling required for bone regeneration. Nevertheless, this property may be advantageous in GBR procedures, where the primary function of the membrane is to act as a barrier rather than as a scaffold for cell ingrowth.

Collagen membranes, particularly non-crosslinked porcine collagen membranes, are traditionally regarded as passive barriers in GBR, preventing soft-tissue ingrowth and stabilizing the underlying clot [23,24]. However, increasing evidence—including our previous work—demonstrates that their porous spongy layer possesses intrinsic osteoconductive properties, permitting cellular invasion, vascular penetration, and early mineral deposition within the matrix [25,26]. It is therefore reasonable to propose that soaking collagen membranes in PPP followed by heat treatment may result in membranes impregnated with heat-coagulated plasma proteins, analogous to Alb-PRF or e-PRF. Importantly, collagen fibrils themselves are sensitive to thermal denaturation. Heating causes native collagen fibrils to lose their characteristic ultrastructure and transition toward gelatin-like behavior at temperatures starting at approximately 40 °C [27,28,29]. This raises the question of whether the thermal processing step used to generate Alb-PRF/e-PRF may inadvertently alter the properties of PPP-soaked collagen membranes intended for GBR.

We therefore hypothesized that applying heat treatment to PPP (hPPP) already absorbed within collagen fibrils would modify membrane function. Specifically, this proof-of-principle study aimed to assess whether such processing induces a directional shift from an osteoconductive, remodelable scaffold toward a more volume-stable, barrier-dominant membrane by comparing native PPP with a single, established heat-treatment protocol.

## 2. Material and Methods

### 2.1. Study Design

**Figure 1:**
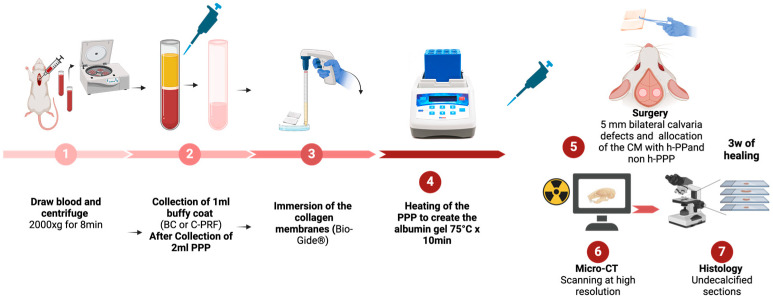
Workflow of experimental design. Schematic representation of the preparation of native and heat-denatured PPP, membrane conditioning, surgical creation of rat calvarial defects, and subsequent micro-CT and histological analyses.

All animal procedures were conducted at the Department of Biomedical Research (DBMR) of the Medical University of Vienna in accordance with institutional and national regulations for animal welfare. The study adhered to the ARRIVE guidelines for the reporting of in vivo experiments. Ethical approval was obtained from the institutional ethics committee and the Austrian Federal Ministry of Education, Science and Research (Approval No. BMWFW-66.009/0399-V/3b/2018). Adult male Sprague–Dawley rats (200–300 g) were randomly allocated to receive calvarial defects treated with collagen membranes (Bio-Gide^®^, Geistlich Pharma, Wolhusen, Switzerland) soaked either with native platelet-poor plasma (PPP) or with PPP subjected to heat denaturation (hPPP). Treatment allocation was performed at the level of individual defects using a random sequence generator and was independent of defect laterality. For the present comparison, one calvarial defect per animal was included in the analysis, while the contralateral defect was allocated to other experimental conditions reported separately. Animals were housed in a controlled environment with ad libitum access to food and water and maintained on a 12 h light/dark cycle. Treatment assignment remained undisclosed to the operating surgeon until the time of membrane placement during defect coverage. Throughout the analytical phase, the examiner (KAA) conducting all assessments was blinded to treatment allocation (Figure 1).

### 2.2. Preparation of Native and Heated PPP

To obtain liquid PPP, venous blood samples were collected by cardiac puncture from two freshly euthanized rats using a 10 mL syringe and plastic tubes without additives (“No Additive” tubes, Greiner Bio-One GmbH, Kremsmünster, Austria). The blood was centrifuged at 2000 g for 8 min using a swing-out rotor (Z306 Hermle Universal Centrifuge, Wehingen, Germany). Collagen membranes were then immersed in the PPP, with one group kept at room temperature while the other half of the membranes was subjected to heating at 75 °C for 10 min using a thermal block (Eppendorf^®^ ThermoMixer^®^, Eppendorf, Germany) and allowed to return to room temperature afterwards. All procedures were performed immediately prior to surgery.

### 2.3. Surgery

Anesthesia was induced by intraperitoneal injection of medetomidine (0.15 mg/kg), midazolam (2 mg/kg), and fentanyl (5 µg/kg). Using a trephine drill (5 mm outer diameter), bilateral 5 mm full-thickness defects were created in the parietal bone. Following randomization at the defect level, each defect was covered with a collagen membrane (6 mm × 6 mm) loaded with either native PPP or hPPP, ensuring a minimum overlap of 1 mm beyond the defect margins in all directions. Treatment allocation remained concealed from the surgeon until the time of membrane placement. Wound closure was performed in two layers using absorbable USP 5-0 sutures. Atipamezole (0.75 mg/kg) and flumazenil (0.2 mg/kg) were administered subcutaneously to reverse sedation. Postoperative analgesia was provided by piritramide administered orally via the drinking water (30 mg piritramide plus 10 mL of 10% glucose solution in 250 mL water). After a three-week healing period, the animals were euthanized by intracardiac administration of an overdose of pentobarbital.

### 2.4. Micro-CT Analysis

Tissue specimens were fixed immediately after retrieval in phosphate-buffered formalin (Roti-Histofix 4%, Carl Roth, Karlsruhe, Germany). High-resolution micro-CT was performed using a μCT 50 system (Scanco Medical AG, Bruttisellen, Switzerland) operated at 90 kV, 200 µA, and a 0.5 mm Al filter. A field of view of 35.2 mm was captured with 1500 projections per 180° with an integration time of 500 ms and reconstructed to an isotropic resolution of 10.3 µm. The reconstructed image stacks were imported into FIJI/ImageJ version 2.16.0/1.54p [30] (National Institutes of Health, Bethesda, MD, USA) and reoriented to align the drilled defect axis with the Z-axis, ensuring central positioning of the defect in all scans. A cylindrical region of interest (ROI), matching the original defect dimensions, was manually defined and applied uniformly to all datasets. Mineralized tissue was segmented using a fixed global threshold of 350 mgHA/cm^3^ to reliably distinguish mineralized from non-mineralized structures. A custom ImageJ macro was implemented to standardize and automate ROI application and segmentation steps. From the segmented volumes, quantitative parameters—including bone volume (BV), defect coverage (Cov%), and trabecular thickness (Tb.Th)—were calculated.

### 2.5. Histological Analysis

After fixation, specimens were dehydrated through a graded ethanol series and embedded in light-curing resin (Technovit 7200 VLC + BPO; Kulzer & Co., Wehrheim, Germany). Resin blocks were processed using a precision cutting and grinding system (Exakt Apparatebau, Norderstedt, Germany) to obtain thin-ground sections through the defect center, oriented parallel to the sagittal suture and perpendicular to the parietal bone. The sectioning plane was guided by micro-CT using 3D visualization software (Amira–Avizo 3D 2021.2; Thermo Fisher Scientific, Waltham, MA, USA). Sections were stained using Levai–Laczko stain consisting of azure II, methylene blue, and pararosaniline, to provide high contrast between mineralized and soft tissues. Stained slides were digitized using a virtual slide scanner (Olympus BX61VS, DotSlide 2.4; Olympus, Tokyo, Japan) equipped with a 20× objective, yielding a final resolution of 0.32 µm/pixel. All digital images were systematically reviewed, and a qualitative descriptive assessment was performed to evaluate tissue organization, membrane behavior, and bone–material interactions.

### 2.6. Statistics

All statistical analyses were performed using R (version 4.0.2). For the present analysis, one calvarial defect per animal was included in each treatment group; therefore, individual defects were treated as independent experimental units. Owing to the small sample size and the non-normal distribution of several outcome variables, comparisons between PPP and hPPP groups were performed using the Wilcoxon rank-sum test (Mann–Whitney U test). Three micro-computed tomography (micro-CT) parameters were analyzed: bone volume (BV), defect coverage (Cov%), and trabecular thickness (Tb.Th). Data are presented as median and interquartile range (IQR). Effect sizes were estimated using the rank-biserial correlation with corresponding 95% confidence intervals. Statistical significance was set at *p* < 0.05. An a priori sample size calculation was performed using G*Power (version 3.1.9.7; Heinrich Heine University Düsseldorf, Düsseldorf, Germany) based on previously published micro-CT data [26]. Using reported BV values (0.85 ± 0.26 mm^3^ for controls and 0.38 ± 0.46 mm^3^ for interventions), with α = 0.05 and 80% power, a minimum of nine animals per group was estimated. Because the present analysis was restricted to one defect per animal and employed non-parametric testing, this calculation should be interpreted as approximate; accordingly, the study is considered exploratory.

## 3. Results

During micro-CT evaluation, a subset of defects in the hPPP group was excluded from quantitative analysis because technical or anatomical factors precluded reliable definition of the ROI. Specifically, in these cases, the collagen membrane was displaced, and mineralized tissue formation occurred predominantly outside the intended membrane-covered defect area, preventing consistent defect delineation. According to the predefined exclusion criteria, a total of *n* = 11 defects in the PPP group and *n* = 9 defects in the hPPP group were included in the quantitative micro-CT analysis.

### 3.1. Micro-CT Assessment

**Figure 2:**
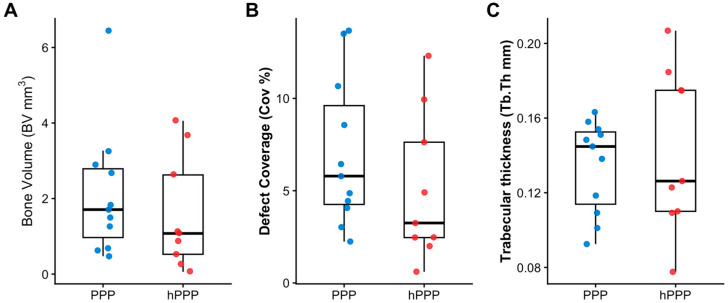
Quantitative micro-CT outcomes comparing collagen membranes with PPP that were left undisturbed (PPP) or underwent thermal treatment (hPPP). (**A**) Bone volume (BV) was numerically higher in the PPP group, but the difference was not statistically significant. (**B**) Defect coverage (Cov%) was numerically higher in the PPP group, with no statistically significant difference between groups. (**C**) Trabecular thickness (Tb.Th) showed overlapping distributions between groups. Each dot represents one defect. Boxplots indicate the median, interquartile range (IQR), and whiskers extend to 1.5× IQR. Group comparisons were performed using the Wilcoxon rank-sum test (Mann–Whitney U test).

**Figure 3:**
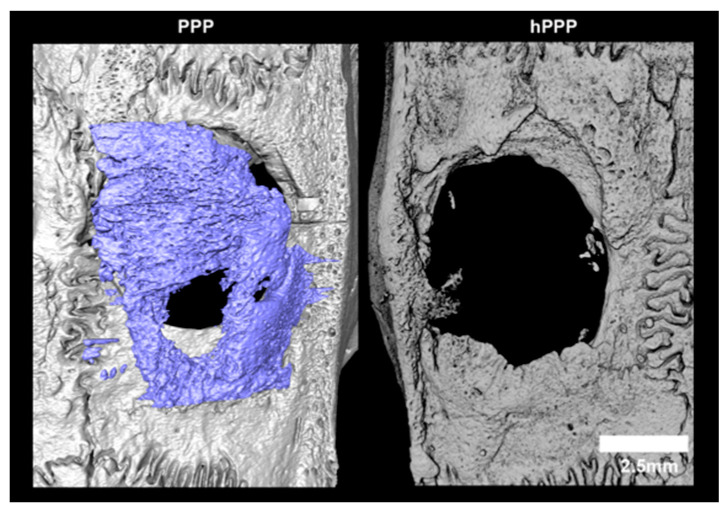
Representative 3D micro-CT reconstructions illustrating defect healing under the two treatment conditions. In defects treated with PPP-coated collagen membranes, abundant mineralized tissue is present and occupies a substantial portion of the defect area (blue segmentation). In contrast, defects treated with heat-treated PPP-coated collagen membranes (hPPP) show limited mineralized tissue within the defect, with the central region remaining largely unfilled. Scale bars are provided for each magnification and apply to all images acquired at the same magnification unless otherwise indicated.

Quantitative micro-CT analysis demonstrated a consistent trend toward greater bone formation in defects treated with collagen membranes soaked with native PPP compared to hPPP membranes (Figure 2). Bone volume (BV) was numerically higher in the PPP group, whereas hPPP-treated defects exhibited lower and more heterogeneous BV values; however, this difference did not reach statistical significance (median [IQR]: 1.71 [0.97–2.79] mm^3^ vs. 1.08 [0.52–2.63] mm^3^; Mann–Whitney U test, *p* = 0.362) (Figure 2A). Similarly, defect coverage (Cov%) tended to be higher in PPP-treated defects (Figure 3), but this difference was also not statistically significant (5.79 [4.25–9.61]% vs. 3.25 [2.46–7.63]%, *p* = 0.224) (Figure 2B). Trabecular thickness (Tb.Th) was comparable between groups, with overlapping distributions and no detectable differences in microarchitectural parameters (0.145 [0.114–0.153] mm vs. 0.126 [0.110–0.175] mm, *p* = 0.543) (Figure 2C). Notably, because the collagen membrane itself was not radiographically distinguishable, micro-CT measurements reflect mineralized tissue within the predefined defect ROI and cannot accurately differentiate mineralization occurring within the membrane from that adjacent to it.

### 3.2. Histological Evaluation of Room Temperature PPP-Coated Collagen Membranes

**Figure 4:**
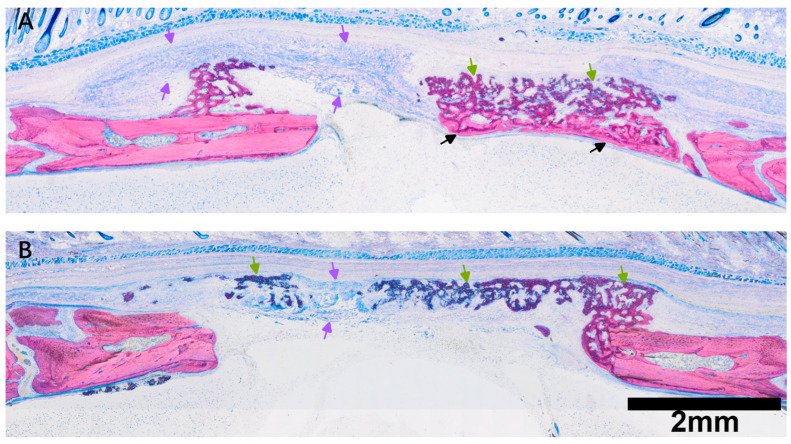
Histological overview of defects treated with room temperature PPP-coated collagen membranes. (**A**) Representative section showing new bone formation at the defect margins and extension of mineralized tissue into the porous layer of the collagen membrane. Hybrid bone structures are observed within the membrane (green arrows), characterized by woven trabeculae intermingled with membrane-derived collagen fibers. Beneath these hybrid structures, more mature lamellar bone (black arrows) is evident along the defect margins and underlying calvarial bone surface. (**B**) Representative PPP-treated specimen showing less pronounced bone ingrowth than in (**A**), while still demonstrating new bone formation extending toward and partially into the membrane (purple arrows). Protein-rich regions are present within the membrane and are located adjacent to areas of new bone formation. Scale bars are shown for each magnification and are representative of all images acquired at the same magnification unless otherwise indicated.

**Figure 5:**
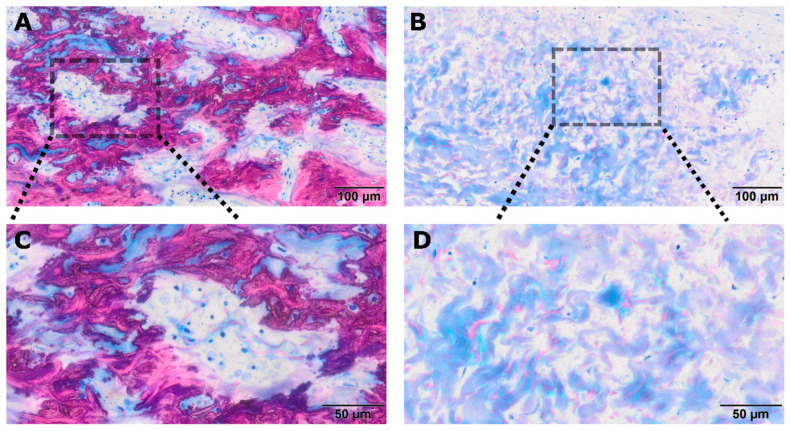
Representative micrographs of room temperature PPP-treated collagen membranes. (**A**) Overview showing hybrid bone formation within the collagen membrane, characterized by irregular trabeculae with embedded collagen fibers, suggestive of passive mineralization. The dashed box indicates the region shown at higher magnification in panel (**C**). (**B**) Corresponding membrane region demonstrating dense albumin-rich areas derived from PPP. The dashed box indicates the area enlarged in panel (**D**). (**C**) Higher magnification of the region highlighted in (**A**), revealing mineralized woven bone with osteoid seams and embedded osteocytes, consistent with active bone formation. (**D**) Higher magnification of the boxed area in (**B**), showing compact protein-entombed collagen fibers with minimal inflammatory infiltrate. In the Levai–Laczko–stained sections, mineralized tissue appears pink to red, whereas albumin- and plasma protein–rich areas stain blue.

Collagen membranes soaked with native PPP showed evidence of early ossification (Figure 4), with mineralized tissue distributed throughout the porous collagen membrane. Newly formed trabeculae exhibited prominent osteoid seams, numerous osteoblast-like cells lining bone surfaces, and embedded osteocytes, consistent with early maturation of woven bone. At higher magnification, hybrid bone structures were observed as previously described [26,31], characterized by the incorporation of mineralized collagen fibers from the membrane into the newly formed bone matrix (Figure 5). In addition, blue-stained regions were present within the membrane; while these areas are presumably protein-rich, their exact composition was not determined in the present study. Overall, these histological observations indicate that native PPP-conditioned collagen membranes were associated with mineral deposition and bone formation within the membrane structure during the early healing period.

### 3.3. Heat-Denatured PPP-Coated Collagen Membranes (hPPP)

**Figure 6:**
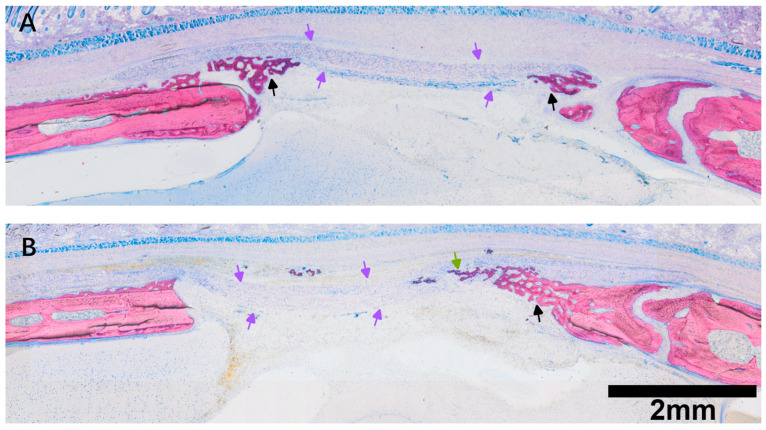
Overview of defects treated with heat-treated PPP-soaked collagen membranes (hPPP). (**A**) Low-magnification section showing largely preserved collagen membrane architecture. Only limited new bone formation (black Arrows) is present, predominantly originating from the defect margins and extending beneath the collagen membrane (purple arrows). (**B**) Bone regeneration remains largely restricted to the periphery of the defect, with sparse, isolated mineralized regions within the defect space (green arrow). The central portion of the membrane appears preserved and shows no evident internal bone ingrowth. Scale bars are shown for each magnification and are representative of all images acquired at the same magnification unless otherwise indicated.

**Figure 7:**
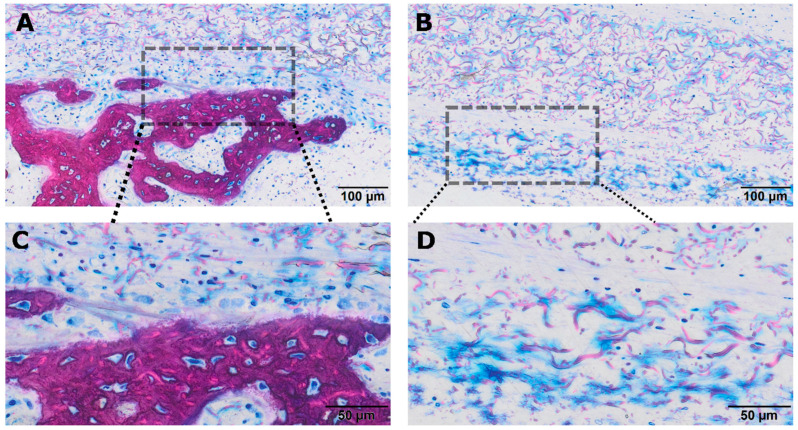
High-magnification histological images of defects treated with heat-treated PPP-soaked collagen membranes (hPPP). (**A**) Mineralized bone at the defect margin showing early hybrid bone attempting to extend toward the heated collagen membrane. The dashed box indicates the area enlarged in panel (**C**). (**B**) Central membrane region displaying collagen fibers with prominent blue albumin aggregates and absence of mineralized tissue. The dashed box indicates the area enlarged in panel (**D**). (**C**) Higher magnification of the hybrid bone interface in panel (**A**), highlighting woven bone, osteoid borders, and numerous embedded osteocytes. (**D**) Higher magnification of the membrane region in panel (**B**), illustrating dense albumin-rich fibrillar clusters with scattered infiltrating cells but no osteoid deposition.

**Figure 8:**
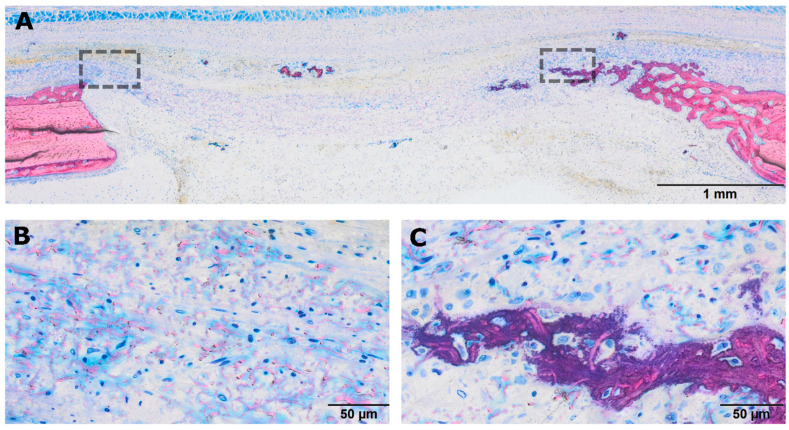
Histological features of defects covered with heat-treated PPP-soaked collagen membranes (hPPP). (**A**) Low-magnification overview showing a preserved collagen membrane architecture with minimal bone formation limited to the defect margins. Dashed boxes indicate the regions shown at higher magnification in panels (**B**,**C**). (**B**) Higher magnification corresponding to the collagen membrane region demonstrating pronounced cellular infiltration, but widely dispersed, unmineralized collagen fibers and dense blue protein aggregates. (**C**) Hybrid bone formation at the periphery but absence of mineralization within the membrane itself.

Heat-denatured collagen membranes previously soaked with PPP exhibited distinct histological features compared with native PPP-treated membranes. After three weeks, the overall membrane structure appeared largely preserved, with minimal evidence of degradation and a spongy layer composed of densely packed, thickened collagen bundles (Figure 6). In central membrane regions, no organized bone formation was observed; collagen fibers displayed diffuse pink mineral staining but lacked osteoid seams or associated bone-forming cells. Patchy dark-blue–stained areas were frequently present within the membrane. New bone formation was predominantly confined to the defect margins, extending from the native calvarial bone, with occasional peripheral hybrid bone formation approaching or partially penetrating the membrane (Figure 7). In addition, numerous cells were observed within hPPP-treated membranes (Figure 8). Overall, these histological observations indicate limited internal bone formation within hPPP-treated membranes during the evaluated healing period, despite the presence of cellular infiltration.

## 4. Discussion

Premature resorption of collagen membranes—long recognized as a key factor limiting the reliability of GBR procedures [31,32]—has motivated the pursuit of clinically practical strategies to extend membrane stability without compromising their native characteristics. The present study was designed to test a directional effect of thermal processing rather than to establish graded or parametric control of membrane properties. In this proof-of-principle comparison, we observed that simultaneous thermal denaturation of PPP-soaked collagen scaffold altered membrane behavior in a rat calvarial defect model. Comparative analysis of native versus thermally treated PPP-conditioned membranes revealed distinct differences in bone formation patterns, cellular infiltration, and qualitative membrane persistence. Together, these findings suggest that thermal processing of both PPP and scaffold can induce a directional shift in membrane behavior within the early healing period evaluated.

Collagen membranes soaked in native PPP supported robust ossification, characterized by mineral deposition throughout the porous network and the formation of hybrid bone structures in which mineralized collagen fibers from the membrane were incorporated into newly formed bone. These observations are consistent with previous reports demonstrating that non-crosslinked collagen membranes possess intrinsic osteoconductive properties, which can be modulated by biological coatings, including plasma fractions [33]. The fibrin- and fibronectin-rich environment provided by native PPP may support early angiogenesis, osteogenic cell recruitment, and matrix remodeling, processes that are critical for bone formation both at defect margins and within the membrane scaffold [34]. Histologically, the presence of osteoid seams and embedded osteocytes within hybrid bone regions is indicative of ongoing bone maturation within the membrane structure. Collectively, these findings reinforce the concept that collagen membranes can function as more than passive barriers, especially when supplemented with biologically active plasma components.

In contrast, membranes soaked in PPP followed by thermal denaturation exhibited markedly different behavior. Thermal treatment at 75 °C for 10 min resulted in a compact, protein-rich matrix that preserved overall membrane architecture but was associated with limited internal bone formation. New bone formation was largely confined to the defect margins, while mineralization within the central membrane regions was minimal. Within the limitations of this model and timepoint, these findings suggest that hPPP shifts the functional behavior of the collagen membrane toward a more barrier-dominant, semi-occlusive construct. The observation that fibroblast-like cells were able to infiltrate hPPP-treated membranes indicates that the barrier was not completely impermeable; however, its capacity to support osteogenic infiltration appeared reduced. At a macroscopic and histological level, this behavior resembles features reported for Alb-PRF and e-PRF matrices, which exhibit prolonged structural persistence at the expense of rapid cellular remodeling [13,19,20,22], although direct compositional or functional equivalence was not assessed in the present study.

An important consideration is that thermal denaturation likely affects both plasma proteins and the collagen fibrils themselves. Native collagen fibrils are sensitive to heat, with temperatures above approximately 40 °C capable of disrupting the triple-helical structure and inducing gelatin-like behavior [27,28,29]. Consequently, the thermal processing used to generate hPPP may have altered the collagen scaffold in addition to coagulating plasma proteins. This combined effect could account for the observed trade-off between enhanced structural persistence and reduced osteoconductive behavior. Histologically, the dense coagulated protein network observed within hPPP-treated membranes may physically limit osteogenic cell penetration while still permitting limited soft-tissue infiltration.

From a mechanistic perspective, these interpretations remain inferential. Native PPP provides a degradable fibrin and fibronectin [35,36] network that is biologically active and permissive to cell migration and vascular ingrowth. Heat denaturation is known to alter protein conformation and reduce susceptibility to enzymatic degradation [17], which may diminish matrix remodeling and delay osteogenic infiltration. However, protease activity, growth factor availability, and ultrastructural matrix changes were not quantified in this study; therefore, mechanistic conclusions should be interpreted cautiously. The occasional presence of isolated mineralized regions within hPPP-treated membranes suggests that passive mineral deposition processes may still occur [25], but these were insufficient to support the extensive hybrid bone formation observed with native PPP.

From a translational standpoint, these findings highlight the importance of plasma processing and thermal exposure in determining collagen membrane behavior during early healing. In contexts where early osteogenesis and membrane remodeling are desired, native PPP-conditioned membranes may be advantageous. Conversely, in situations where space maintenance and delayed membrane degradation are priorities, thermally treated PPP-conditioned membranes may warrant further investigation. Importantly, these observations should not be interpreted as clinical recommendations but rather as a basis for future studies aimed at defining how controlled thermal processing influences membrane performance under different regenerative conditions.

Several limitations should be acknowledged. First, the study was restricted to a single early healing timepoint (21 days) and a limited number of defects, reducing statistical power—particularly for non-parametric analyses—and confining interpretation to early osteogenic events. Accordingly, differences in bone volume and defect coverage should be interpreted as directional trends rather than definitive quantitative effects. Second, the rat calvarial defect model is non-load-bearing and does not fully replicate the mechanical environment encountered in clinical GBR. Third, molecular and mechanical analyses were not performed, limiting insight into the biological and physical mechanisms underlying the observed effects. In the present design, native PPP-conditioned membranes served as the biological control, and the impact of thermal processing was assessed by comparison with hPPP-loaded membranes. Untreated membranes or heat-treated membranes without plasma were not included, as the study aimed to isolate the effect of plasma denaturation within a fixed collagen scaffold. Finally, only one heat-treatment protocol was evaluated; intermediate temperatures and exposure times were beyond the scope of this proof-of-principle study but represent an important direction for future work.

## 5. Conclusions

In summary, thermal denaturation of PPP, together with heat-induced modification of collagen fibrils, was associated with a qualitative shift in membrane behavior during early healing, characterized by reduced internal osteogenesis and increased structural persistence. Native PPP, by contrast, preserved both plasma bioactivity and collagen permissiveness, supporting bone formation throughout the membrane. These complementary behaviors support the concept that plasma processing and thermal exposure influence the balance between osteogenic support and barrier function in collagen membranes, providing a foundation for future studies aimed at rationally altering GBR biomaterials.

## Data Availability

The original contributions presented in this study are included in the article. Further inquiries can be directed to the corresponding author.
